# Patients’ Willingness to Share Information in Online Patient Communities: Questionnaire Study

**DOI:** 10.2196/16546

**Published:** 2020-04-01

**Authors:** Panpan Zhu, Jiang Shen, Man Xu

**Affiliations:** 1 College of Management and Economics Tianjin University Tianjin China; 2 Business School Nankai University Tianjin China

**Keywords:** online patient community, information sharing, willingness to share, questionnaire, structural equation

## Abstract

**Background:**

Online patient communities provide new channels for users to access and share medical information. In-depth study of users’ willingness to share information in online patient communities is of great significance for improving the level of information sharing among the patient community and the long-term development of communities.

**Objective:**

The aim of this study was to build a model of factors affecting patients’ willingness to share medical information from the perspective of both positive and negative utilities. Specifically, we aimed to determine the influence of online information support and privacy concerns, as well as the moderating effect of disease severity and information sensitivity of different patients on their willingness to share.

**Methods:**

Data from 490 users with experience in online patient communities were collected through a questionnaire survey, and structural equations were applied to empirically verify the model hypotheses.

**Results:**

Privacy concerns negatively affected the patients’ willingness to share information (*P*<.001), whereas online information support positively affected patients’ willingness to share information (*P*<.001), and information sensitivity negatively moderated the impact of online information support on sharing willingness (*P*=.01). Disease severity positively moderated the impact of privacy concerns on sharing willingness (*P*=.05). However, the hypotheses that information sensitivity is a negative moderator and disease severity is a positive moderator of the impact of privacy concerns on sharing willingness could not be supported.

**Conclusions:**

To improve the level of user information sharing, the online patient community should design a safe user registration process, ensure the confidentiality of information, reduce the privacy concerns of users, and accurately identify the information needs of patients to provide personalized support services.

## Introduction

### Background

Social media is increasingly being used by health services to encourage the growth of social support networks among people with health-related issues [1,2]. Through participation on different platforms, patients are able to provide and receive social support in various ways, without the typical barriers and constraints experienced by this population [3]. In this way, problems of information asymmetry, as well as the shortages and uneven distribution of medical resources can be alleviated [4,5]. Jackson et al [6] studied the characteristics of patients who shared medical records with others, and found that participants who shared medical records were more likely to experience better health management. To obtain information support from other patients in the community, users need to share information on personal medical treatments, medication records, or medical diagnosis. However, disclosing such information may also have serious adverse consequences [7]. In the face of risky decisions about online information demands and privacy disclosure, patients may choose whether to share such personal information according to their judgment on the possible benefits and losses. Therefore, in-depth study of users’ information-sharing willingness and the decision-making mechanisms of online patient communities is of great significance to the improvement of information sharing among patient communities and the long-term development of these communities.

Most of the existing research on patient information sharing focuses on the impacts of sharing willingness from the perspective of the value brought by online communities [8-10]. However, users usually consider both the gains and risks associated with information sharing when making decisions about to whether they should share information [11]. In this context, we here establish a model of factors affecting patients’ willingness to share medical information combined with utility theory, which considers both the influence of online information support (OIS) and privacy concerns on patients’ willingness to share information (WSI) from the perspectives of both the positive and negative utilities brought by information sharing to users. The information-sharing decisions of online community users depend not only on the community environment (ie, whether the community provides OIS and whether the community environment can dispel users’ privacy concerns) but also on the personal attributes of each community user (ie, health status and sensitivity to information). If patients are not sensitive to the positive utility of information sharing but are sensitive to the negative utilities (ie, afraid of the risks), the probability that they will choose not to share information is high, whereas if patients are not sensitive to the risks of information sharing but are very sensitive to the positive utilities, the possibility of sharing information is high. Therefore, the main contribution of this paper is to introduce patients' information sensitivity and health status into the model as moderating variables to explore the moderating effect of such individual patient attributes on information-sharing intention.

### Literature Review and Hypotheses

#### Information-Sharing Behaviors in Online Patient Communities

Information sharing refers to the process during which individuals share the information they own with other members in the community in various forms and routes [12]. In most cases, members of online patient communities are patients troubled by different diseases [13]; therefore, they have relatively strong empathy. Their similar situations enable them to easily obtain both the emotional and information support they need while relating to the feelings of other members of the community during the process of communication [14]. Combined with the features of online patient communities, shared information can be classified into general health information and special health information. General health information refers to common medical and health information, which is usually open to the public and can be easily obtained by the masses, such as information on doctors and hospitals, prices of medicines, and features of a specific disease. Special health information is usually concerned with privacy, which refers to personal empirical information, including medical records, treatment, and personal health conditions [15].

#### Utility Theory

Utility theory is often adopted when individuals make decisions [16]. Utility is defined as the degree to which a user’s psychological expectation of a certain factor is satisfied or realized [17]. This judgment is completely controlled by personal preferences, interests, or the subjective consciousness: the higher the satisfaction degree, the larger the utility, and vice versa [18]. The criterion is maximizing utility by weighing costs and benefits [18], and is considered the ultimate expression of reason and egotism, reflecting the decision maker’s attitude toward risks. In the utility theory formula *U_D_*=*f*(*B,C*), *C* refers to the potential risk of privacy disclosure caused by information sharing, which is a variable indicating negative utility to users; *B* refers to the positive utility obtained by users, which mainly includes the OIS that patients receive; and *D* refers to the decision of users on whether or not to participate in information sharing after judging the positive and negative utilities brought by this behavior.

Information sharing creates value for users but also invites risks. Different individuals have different sensitivities to personal medical health information; such difference in sensitivity could result in different concerns regarding information sharing, thereby affecting their WSI. In addition, different patients have different physical health conditions. If the disease of a patient is severe, the patient may be urgently seeking OIS, which could weaken relative concerns regarding privacy. In this case, the patient is more likely to acquire the necessary information by positively participating in information exchange in communities.

**Figure 1:**
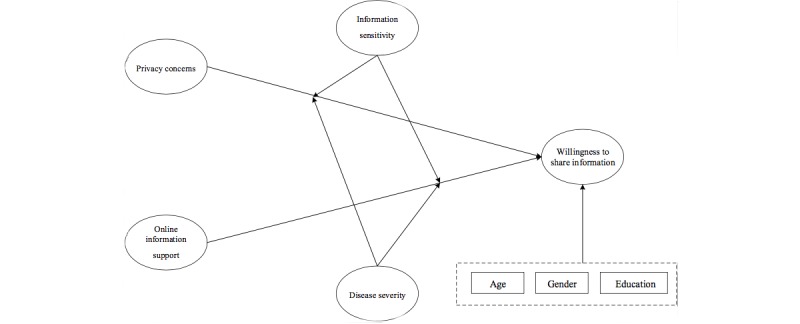
Model of information-sharing intentions of patients in online health communities.

Based on the above analysis, we established an influence model of OIS and privacy concerns for patients’ WSI (Figure 1). The moderating functions of patients’ information sensitivity and disease severity on the WSI were also considered. Specifically, we consider two problems in this model structure: (1) the influence of OIS and privacy concerns on the willingness to share medical information, and (2) the functions of information sensitivity and disease severity as moderating variables.

In this model, the information-sharing intention of patients is the dependent variable, privacy concerns and OIS are independent variables, and information sensitivity and disease severity are moderator variables. That is, privacy concerns and OIS are the main factors affecting patients’ intentions to share medical information, the effects of which are regulated by disease severity and information sensitivity. Moderator variables with increasing negative effects (ie, negative moderator influence factors) affect sharing intentions, whereas moderator variables with decreasing negative effects (ie, positive moderator influence factors) also affect sharing willingness. The control variables include gender, age, and education level. Along with the different social roles and characteristics between genders, feelings and behaviors can differ according to gender regarding the same issue [19]. As a result, gender may influence information-sharing intentions. Age may also have effects on information-sharing intentions since age influences physical condition and life experience, which can result in quite different opinions and feelings about the same issue [20]. With respect to education level, higher education levels are associated with stronger judgment and cognitive competence; thus, behavioral patterns may differ across this characteristic as well [21] to ultimately affect the information-sharing intentions of online health community users.

### Research Hypotheses

**Figure 2:**
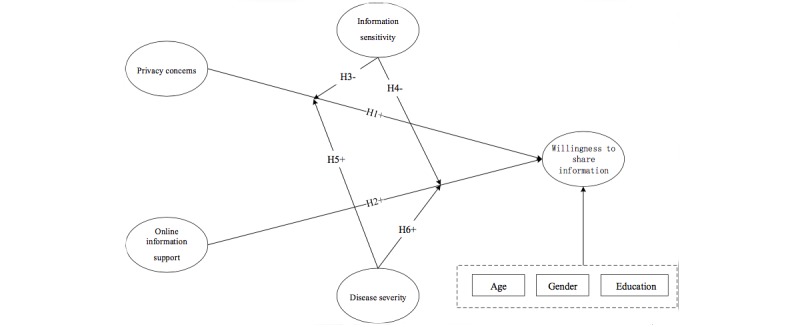
Research hypotheses on online information-sharing willingness.

Based on the above theoretical model, six hypotheses are proposed. As shown in Figure 2, the hypotheses are composed of two parts: hypotheses of direct effects and hypotheses of moderation effects.

#### Hypotheses of Direct Effects

##### Relationship Between Online Information Support and Patients' Willingness to Share Information

In an online patient community, patients conduct information sharing to acquire medical assistance and personalized experience information [22]. In a study of two online communities, Hargreaves et al [23] found that patients share experiences, information, and emotions, and receive empathetic support in a supportive and warm community atmosphere. Based on the theory of planned behavior and the theory of technology acceptance, Zhou [24] found that social returns and perceived usefulness can determine the intentions to share personal health information. On the basis of a technology acceptance model, Zhang [25] found that users’ usefulness of acquiring perceptual information from virtual communities can affect their information-sharing behaviors. On the basis of social exchange theory, Gui and Hu [26] found that mutual benefits can significantly affect intentions to share health knowledge by conducting empirical analyses. Thus, research employing various theoretical perspectives has demonstrated that OIS has positive impacts on the information-sharing intentions of patients; in other words, support or help from other community members strengthens the intention to share information.

Thus, the following hypothesis is presented:

H1: OIS has positive impacts on patients’ WSI.

##### Relationship Between Privacy Concerns and Patients' Willingness to Share

As electronic health records become ubiquitous in the health care industry, privacy breaches are increasing [27]. In an online patient community, patients can predict but not fully control these potential risks, thereby reducing their WSI [28]. Privacy concerns are one of the major factors affecting attitudes toward exchanging electronic health information [29,30]. The privacy of patients is prominent in health information exchange discussions, given that their potentially sensitive personal health information may be electronically shared for various health care purposes [31]. Based on a grounded theory, Holloway et al [32] found that issues of privacy and confidentiality were paramount to information sharing. In an empirical study, Zhang et al [33] found that privacy concerns have obvious negative impacts on the attitudes of online patient community users regarding information disclosure. The main objectives of different methods of health information exchange are to reduce privacy concerns [34]. Frost et al [35] studied the information-sharing preferences of cancer patients in the community and found that poor online privacy experience, age, and health status negatively affected the WSI.

Thus, the following hypothesis is presented:

H2: Privacy concerns have negative impacts on patients’ WSI.

##### Hypotheses of Moderation Effects

Information sensitivity, which is closely related to privacy concerns, refers to the extent of concern of individuals regarding information in a specific environment: the greater the extent of concern, the more sensitive the information [36]. Medical information also includes personal information that is not directly related to personal health, such as population statistics (ie, name and date of birth) and information used to identify a patient’s personal identity (ie, ID card number, family address, private phone number, financial information, and insurance status). In addition, such information includes details that may help in making a correct diagnosis and adopting proper treatment measures, such as the cause for seeking medical advice, symptoms, medical history of family members, drug allergy history, and medical history, as well as disease data arising from diagnosis and treatment, such as examination results, progress notes, operation consent, anesthesia consent, operation records, and anesthesia records. Since most of this information involves personal privacy, online sharing causes concern regarding privacy disclosure: the more sensitive the information, the greater the privacy concerns.

Many people believe that if sensitive information is shared or disclosed, it will have bad consequences or even cause damage [37]. Consumers’ intentions to disclose personal information are closely related to the sensitivity of such information [38]. Milne [39] found that the validity of information changes with the sensitivity of such information when collecting personal information from users. According to Okazaki et al [40], information sensitivity increases privacy concerns. Information sensitivity is frequently used as the prepositive variable of privacy concerns [41]. In a network environment, privacy concerns cause a reduction in the publishing of information by online users, whereas information sensitivity causes concerns regarding private information [42]. In this study, the information sensitivity of patients was used as a moderator variable to explain the behavioral intentions of patients regarding sharing information in online patient communities. The users’ privacy concerns during information sharing will be more serious with respect to more sensitive medical information, thereby affecting sharing behavioral decisions [43].

Thus, the following hypotheses are presented:

H3: Information sensitivity negatively moderates the impact of privacy concerns on patients' WSI.

H4: Information sensitivity negatively moderates the impact of OIS on patients' WSI.

Disease severity refers to the users' perception of their own health status; that is, a subjective perception of whether one’s health is good or bad will affect their information-sharing intentions. Tisnado et al [44] studied the impacts of demographic characteristics and individual health on the concordance between medical records and health information sharing. Patients who considered their health to be poor were more sensitive about sharing their personal health information compared with others; these patients worried that they may face uncontrollable risks if their medical and health information were to be disclosed [45]. Health conditions as perceived by online patient community users translate into privacy concerns regarding their medical and health information, which ultimately affect their intentions to share this information [46]. In online patient communities, the priority of patients is to improve their health, interact with other community users, and seek medical assistance. The levels of participation and activeness in the community also vary according to diversified health conditions. When they are physically healthy, they need not consult doctors or tell others about their medical treatment experiences; considering the privacy of medical information, their intentions to share relevant medical and health information may be reduced. When they are physically unhealthy, they urgently need to acquire the information support and assistance provided by online communities to improve their health [47]. However, the precondition is that to receive this assistance, they will need to illustrate their health condition to relevant helpers. Therefore, they have to actively share medical information, and thus their intentions to share relevant information increase. Consequently, the disease severity perceived by online patient community users may affect their intentions in sharing personal medical information by regulating their privacy concerns and OIS demands. In this study, disease severity was used as a moderator variable to moderate the relationship between privacy concerns and OIS demand intensity when both sharing and intending to share health and medical information so as to verify the impact on sharing behavioral patterns.

Thus, the following hypotheses are presented:

H5: Disease severity positively moderates the effect of privacy concerns on patients' WSI.

H6: Disease severity positively moderates the effect of OIS on patients' WSI.

## Methods

### Questionnaire Design

The questionnaire was composed of three parts: Part 1 reflects the experience of the respondents in sharing medical information with any online health community (if such experience is available), Part 2 reflects the objective cognition of the respondents regarding online medical services, and Part 3 mainly concerns the respondents’ basic information and medical history. The medical information sharing intentions, disease severity, and perceived health information sensitivity were measured according to the method of Bansal et al [48], and privacy concerns and OIS were measured according to the method of Liang et al [49]. A typical 7-point Likert scale was used to score opinions and conclusions, with dimensions of “quite disagree,” “disagree,” “basically disagree,” “neutral,” “basically agree,” “agree,” and “quite agree”, respectively. The specific scale is shown in Table 1.

**Table 1:** Measurement scale.

Component and measurement number		Scale	
**Willingness to share (WSI)**			
	WSI1		I am willing to provide my medical health information to online medical websites to get proper treatment.
	WSI2		I agree to let online medical websites use my medical health information.
	WSI3		I do not think it is improper if my medical health information is revealed to online medical websites.
	WSI4		It is highly probable that I will publish my medical health information on some online medical websites.
**Privacy concerns (PC)**			
	PC1		I worry that my medical health information posted online may be misused.
	PC2		I worry that other people can see my medical health information online.
	PC3		In my opinion, personal medical health information is more important than other information.
	PC4		I worry that my medical health information posted online will be used by others in an unexpected manner.
**Disease severity (DS)**			
	DS1		My body is seldom subject to long-term pain and discomfort.
	DS2		I have no chronic disease.
	DS3		My overall health is good.
**Online information support (OIS)**			
	OIS1		When I need assistance related to health issues, anyone from an online medical website can provide relevant advice.
	OIS2		When I encounter any health problem, anyone from an online medical website can provide relevant information to help me overcome the problem.
	OIS3		Anyone from an online medical website will help me find the cause and provide relevant suggestions for any health problem I might have.
**Information sensitivity (IS)**			
	IS1		Medicine taken
	IS2		Current health condition
	IS3		Medical history
	IS4		Process of seeking medical advice

### Data Collection

To guarantee the validity of the questionnaire design and content, many experts and some respondents were invited to evaluate each question in the questionnaire. An internal test was conducted within a small range before issuing these questionnaires. After confirming that there were no errors, the questionnaires were issued through wjx.cn, an online platform for issuing questionnaires. Those who had experience sharing information in online health communities were invited to fill in these questionnaires. A total of 531 copies were collected, 41 of which were incomplete, unreasonable, or unqualified, leaving a total of 490 valid responses.

### Data Analysis

The statistical description of the correlated variables is shown in Table 2.

**Table 2:** Statistical description of variables.

Variables		Sample, n (%)
**Gender**		
	Men	168 (34.3)
	Women	322 (65.7)
**Age (years)**		
	≤20	3 (0.6)
	21-30	216 (44.1)
	31-40	243 (49.6)
	41-50	28 (6)
	51-60	0 (0)
	>60	0 (0)
**Education level**		
	Middle school and below	0 (0)
	High school	10 (2)
	College	43 (9)
	Bachelor	357 (72.9)
	Master	68 (14)
	Doctorate and above	12 (3)
**Duration of using the internet (years)**		
	≤1	0 (0)
	2-4	40 (8)
	5-7	123 (25.1)
	8-10	171 (34.9)
	>10	156 (31.8)
**Duration of using online medical websites (years)**		
	≤1	93 (19)
	2-3	278 (56.7)
	4-5	101 (20.6)
	6-7	15 (3)
	>7	3 (0.6)
**Annual frequency of getting ill**		
	≤1	88 (18)
	2-3	291 (59.4)
	4-6	93 (19)
	7-10	15 (3)
	>10	3 (0.6)

## Results

### Measurement Model Testing

#### Reliability Test

The inspection criteria for the reliability and validity tests were the load levels of the corresponding components, common factor loading, Cronbach alpha, composite reliability, and average variance extracted (AVE). The desired values for these indices [50] are shown in Table 3.

The measurement results for the data factor loading coefficients are shown in Table 4.

The factor loading measurements <0.7 in the questionnaire were deleted, with those remaining reaching the criteria. The results of the modified Smart PLS data analysis are shown in Table 5.

As shown in Table 5, Cronbach alpha for each component was larger than 0.77, indicating that the reliability for each question in the research design was good. The composite reliability coefficient of each component was larger than 0.78, the AVE for each component was greater than 0.57, and all components could be aggregated effectively.

**Table 3:** Desired values for the reliability test.

Evaluation index	Critical value
Factor loading coefficient	>0.70
Cronbach alpha	>.70
Composite reliability coefficient	>0.70
Average variance extracted	>0.50

**Table 4:** Factor loading coefficients for all variables.

Component and scale		Factor loading coefficient
**Privacy concerns (PC)**		
	PC1	0.934
	PC2	0.922
	PC3	0.767
	PC4	0.938
**Online information support (OIS)**		
	OIS1	0.836
	OIS2	0.848
	OIS3	0.808
**Information sensitivity (IS)**		
	IS1	0.671
	IS2	0.951
	IS3	0.850
	IS4	0.756
**Disease severity (DS)**		
	DS1	0.717
	DS2	0.981
	DS3	0.776

**Table 5:** Reliability of test results.

Variable	AVE^a^	Composite reliability	*R* ^2^	Cronbach alpha	Communality	Redundancy
Age	1	1	N/A^b^	1	1	N/A
DS^c^	0.574	0.785	N/A	0.854	0.574	N/A
EDU^d^	1	1	N/A	1	1	N/A
Gender	1	1	N/A	1	1	N/A
IS^e^	0.631	0.869	N/A	0.846	0.631	N/A
OIS^f^	0.690	0.870	N/A	0.775	0.690	N/A
PC^g^	0.731	0.913	N/A	0.889	0.731	N/A
WSI^h^	0.654	0.883	0.331	0.823	0.654	0.039

^a^AVE: average variance extracted.

^b^N/A: not applicable.

^c^DS: disease severity.

^d^EDU: education level.

^e^IS: information sensitivity.

^f^OIS: online information support

^g^PC: privacy concerns.

^h^WSI: willingness to share information.

#### Validity Test

In general, two measurement indices are used for the validity test for each component: the discriminant validity test requires that the correlation coefficient between two components be less than its AVE value, and the convergent validity test requires that the load value at each proposed measured variable be larger than that loaded at any other measured variable in cross-factor loading. Table 6 shows the correlation coefficients used for measuring discriminant validity; diagonal elements contain the arithmetic square root of AVE, and nondiagonal elements contain the correlation coefficient for each corresponding component. Based on these correlations, the data have favorable discriminant validity by satisfying relevant requirements. Table 7 further shows that the factor loading loaded at the corresponding component was larger than that loaded at any other latent variable, demonstrating quite good convergent validity between two components.

**Table 6:** Correlation coefficients.

Variable	Age	DS^a^	EDU^b^	Gender	IS^c^	OIS^d^	PC^e^	WSI^f^
Age	1	—^g^	—	—	—	—	—	—
DS	–0.120	1	—	—	—	—	—	—
EDU	–0.204	–0.085	1	—	—	—	—	—
Gender	–0.044	–0.021	–0.100	1	—	—	—	—
IS	–0.158	–0.038	0.093	0.0351	1	—	—	—
OIS	0.123	0.209	–0.116	0.032	0.132	1	—	—
PC	–0.179	0.079	0.092	–0.041	0.466	0.083	1	—
WSI	0.254	0.158	-0.009	0.043	–0.129	0.436	–0.267	1

^a^DS: disease severity.

^b^EDU: education level.

^c^IS: information sensitivity.

^d^OIS: online information support.

^e^PC: privacy concerns.

^f^WSI: willingness to share.

^g^Not applicable.

**Table 7:** Cross-factor loadings.

Variable	Age	DS^a^	EDU^b^	Gender	IS^c^	OIS^d^	PC^e^	WSI^f^
Age	1	–0.120	–0.204	–0.044	–0.158	0.123	–0.179	0.254
DS1	–0.164	0.410	–0.031	0.029	0.149	0.136	0.308	–0.037
DS2	–0.137	0.980	–0.089	–0.009	–0.009	0.214	0.123	0.144
DS3	–0.161	0.771	–0.018	–0.031	0.064	0.189	0.237	0.020
EDU	–0.204	–0.085	1	–0.100	0.093	–0.116	0.092	–0.009
Gender	–0.044	–0.021	–0.100	1	0.035	0.032	–0.041	0.043
IS1	–0.090	–0.071	0.080	–0.017	0.570	–0.008	0.243	0.009
IS2	–0.152	–0.059	0.049	–0.017	0.950	0.080	0.380	–0.152
IS3	–0.138	0.015	0.153	0.117	0.851	0.217	0.487	–0.063
IS4	–0.109	–0.029	0.104	0.064	0.757	0.090	0.430	–0.047
OIS1	0.080	0.101	–0.108	0.086	0.088	0.835	0.047	0.369
OIS2	0.090	0.222	–0.047	0.069	0.074	0.848	0.026	0.358
OIS3	0.137	0.199	–0.132	–0.076	0.168	0.809	0.133	0.361
PC1	–0.100	0.056	0.094	–0.054	0.447	0.102	0.934	–0.221
PC2	–0.243	0.147	0.044	–0.103	0.420	0.070	0.922	–0.271
PC3	–0.125	0.092	–0.005	0.015	0.285	0.202	0.570	0.003
PC4	–0.143	0.011	0.124	0.047	0.438	0.065	0.937	–0.247
WSI1	0.179	0.115	–0.009	0.110	0.041	0.516	–0.048	0.733
WSI2	0.184	0.185	–0.076	0.050	–0.185	0.293	–0.245	0.837
WSI3	0.260	0.161	0.025	–0.016	–0.163	0.268	–0.283	0.820
WSI4	0.195	0.053	0.025	–0.009	–0.123	0.315	–0.297	0.841

^a^DS: disease severity.

^b^EDU: education level.

^c^IS: information sensitivity.

^d^OIS: online information support.

^e^PC: privacy concerns.

^f^WSI: willingness to share

### Results of Structural Model Testing

After fully verifying the reliability and validity of the questionnaire, we next analyzed the structural model to test the various proposed hypotheses based on the following values: (1) the path coefficient between each variable, (2) the significance of the *t* value, and (3) the degree of *R*^2^ to which the dependent variable is interpreted. In line with the research model, significance verification was conducted in two steps: the first step assessed the significance of the influencing factors among the major components (namely, verification of the basic model), and the second step examined the effect of the regulated variable added to the model. The results are shown in Table 8.

**Table 8:** Verification of the basic model.

Hypothesis	t_485_	*P* value	Supported
H1: online information support has positive impacts on patients’ willingness to share information**.**	5.77	<.001	Yes
H2: privacy concerns have negative impacts on patients’ willingness to share information	3.41	<.001	Yes
H3: information sensitivity negatively moderates the impact of privacy concerns on patients' willingness to share information.	0.96	.34	No
H4: information sensitivity negatively moderates the impact of online information support on patients’ willingness to share information.	2.58	.01	Yes
H5: disease severity positively moderates the effect of privacy concerns on patients’ willingness to share information.	1.96	.05	Yes
H6: disease severity positively moderates the effect of online information support on patients’ willingness to share information.	0.68	.50	No

## Discussion

### Principal Findings

This study showed that privacy concerns have a negative impact on users’ WSI, and the OIS provided by online communities has a positive impact on the users’ WSI. The hypothesis that information sensitivity negatively regulates the impact of privacy concerns on WSI was not supported. The indirect impact of information sensitivity on WSI mainly occurs through negative regulation of the OIS offered by communities. This phenomenon may be caused by the platform’s privacy protection mechanism. In most cases, community platforms include certain user privacy protection measures to protect users’ personal data from being leaked. When patients participate in online communication, most of their personal information is blocked and invisible to others. Despite the involvement of relatively sensitive information, users can be free from concerns regarding disclosure of their personal identity, which greatly diminishes the effect of information sensitivity on privacy concerns. Although the privacy protection measures of the community platform dispel the inhibition effect of privacy concerns caused by patients’ information sensitivity on WSI to some degree, information sensitivity still negatively regulates the positive effect of OIS on WSI. If patients are in poor health and need to obtain information from other patients in the community, their privacy concerns will be reduced to a certain extent. The mental inhibition factors of information sharing are reduced, while the weakened privacy concerns further increase the willingness to participate in information sharing. In summary, these results suggest that a community platform should make all efforts to desensitize the information shared by users while providing effective information support for users so as to reduce the negative effects of information sharing, increase the benefits of information sharing, and improve the overall information-sharing level of community users.

### Theoretical and Practical Significance

The information-sharing intention model established in this study enriches and improves theoretical knowledge in the following aspects. Primarily, utility theory was applied to study users’ WSI in online patient communities. Previous studies on users’ willingness to share have mainly focused on aspects of motivation, mutual benefits, social exchange, and the like. Yet, by combining the features of users’ information sharing in online patient communities, this study adopted utility theory to probe users’ information-sharing decisions under the condition that both privacy concerns and information demands exist from the user’s perspective. In addition, based on the characteristics of shared information, we further analyzed the information sensitivity and the adjustment effect of the patients' own perceived health status on their WSI. Under the combined effect of the above factors, assessment of users’ WSI in this manner expands the application of utility theory to studies on information-sharing behaviors, thereby laying the foundation for further development of utility theory.

This research also has practical significance. In view of the mechanism of users’ WSI, online service operators should establish a reasonable mechanism to ensure the security and confidentiality of users’ medical information through reasonable and safe user registration processes and appropriate protection measures, thereby minimizing users’ privacy concerns during the process. Moreover, the patients’ health status information should be obtained to accurately identify individual information requirements, so that the platform can offer personalized support services while providing appropriate guidance and incentives, thereby improving the information-sharing level of community users.

### Limitations

As with all empirical studies, this study also has limitations. First, online patient communities comprise a large group of users distributed in various cities across the country. Although the data of this study were obtained from the Internet, the sample size is negligible compared with the enormous group of community users. In addition, the sample data were all obtained from China (without considering a potential Western cultural background), which may affect the conclusions of the study to a certain extent. Second, the subject of information sharing in online patient communities is necessarily “human,” and thus the sharing behavior strategy is inclined to be affected by multiple factors. Moreover, with constant changes in other users’ sharing behaviors, future studies could focus more on the dynamic adjustment of users’ sharing strategies, thereby providing a deeper understanding of the entire sharing process.

### Conclusions

This study established a model to probe the factors influencing decision making on patients’ WSI from the perspectives of both positive and negative utilities. The results confirm the positive and negative effects of OIS and privacy concerns on users’ WSI. In addition, we showed that information sensitivity negatively moderates the impact of OIS on sharing willingness, whereas disease severity positively moderates the impact of privacy concerns on sharing willingness. Therefore, the management of the platform should focus on desensitizing the information shared by users, while providing effective information support for users to reduce the negative effects of information sharing, increase the benefits of information sharing, and improve the information-sharing level of community users. This research can help community platform operators to clarify the behavioral mechanism of users’ independent disclosure of medical information, provide theoretical guidance for operators to stipulate effective measures that encourage users to voluntarily disclose medical information, and offer a reference to improve the operators’ user management of online patient community services.

## Abbreviations

AVE: average variance extracted
DS: disease severity
EDU: education level
IS: information sensitivity
OIS: online information support
PC: privacy concerns
WSI: willingness to share information

## References

[1] Meng F, Guo X, Peng Z, Zhang X, Vogel D (2019). The routine use of mobile health services in the presence of health consciousness. Electron Commer R A.

[2] Ellway D, Reilly R, Le Couteur Amanda, Ward James (2019). Exploring how people affected by methamphetamine exchange social support through online interactions on Facebook: Content Analysis. JMIR Ment Health.

[3] Stetten NE, LeBeau K, Aguirre MA, Vogt AB, Quintana JR, Jennings AR, Hart M (2019). Analyzing the Communication Interchange of Individuals With Disabilities Utilizing Facebook, Discussion Forums, and Chat Rooms: Qualitative Content Analysis of Online Disabilities Support Groups. JMIR Rehabil Assist Technol.

[4] Matthew-Maich N, Harris L, Ploeg J, Markle-Reid M, Valaitis R, Ibrahim S, Gafni A, Isaacs S (2016). Designing, Implementing, and Evaluating Mobile Health Technologies for Managing Chronic Conditions in Older Adults: A Scoping Review. JMIR mHealth uHealth.

[5] Klein DM, Fix GM, Hogan TP, Simon SR, Nazi KM, Turvey CL (2015). Use of the Blue Button Online Tool for Sharing Health Information: Qualitative Interviews With Patients and Providers. J Med Internet Res.

[6] Jackson SL, Mejilla R, Darer JD, Oster NV, Ralston JD, Leveille SG, Walker J, Delbanco T, Elmore JG (2014). Patients Who Share Transparent Visit Notes With Others: Characteristics, Risks, and Benefits. J Med Internet Res.

[7] Fan K, Jiang W, Li H, Yang Y (2018). Lightweight RFID Protocol for Medical Privacy Protection in IoT. IEEE Trans Ind Inf.

[8] Yang H, Ju X (2017). The effects of social support and individual goal on health condition. J Manag Sci.

[9] Zhang X, Liu S, Deng Z, Chen X (2017). Knowledge sharing motivations in online health communities: A comparative study of health professionals and normal users. Comput Hum Behav.

[10] Oh S (2011). The characteristics and motivations of health answerers for sharing information, knowledge, and experiences in online environments. J Am Soc Inf Sci.

[11] Zhang X, Guo X, Wu Y, Lai K, Vogel D (2017). Exploring the inhibitors of online health service use intention: A status quo bias perspective. Inf Manag.

[12] Vaala S, Lee J, Hood K, Mulvaney S (2018). Sharing and helping: Predictors of adolescents? willingness to share diabetes personal health information with peers. J Am Med Informatics Assoc.

[13] Nambisan P, Gustafson DH, Hawkins R, Pingree S (2015). Social support and responsiveness in online patient communities: impact on service quality perceptions. Health Expect.

[14] McCaig D, Elliott MT, Siew CS, Walasek L, Meyer C (2019). Profiling Commenters on Mental Health–Related Online Forums: A Methodological Example Focusing on Eating Disorder–Related Commenters. JMIR Ment Health.

[15] Lognos B, Carbonnel F, Boulze Launay I, Bringay S, Guerdoux-Ninot E, Mollevi C, Senesse P, Ninot G (2019). Complementary and Alternative Medicine in Patients With Breast Cancer: Exploratory Study of Social Network Forum Data. JMIR Cancer.

[16] Dhami S, Wei M, al-Nowaihi A (2019). Public goods games and psychological utility: Theory and evidence. J Econ Behav Org.

[17] Le LK, Sanci L, Chatterton ML, Kauer S, Buhagiar K, Mihalopoulos C (2019). The Cost-Effectiveness of an Internet Intervention to Facilitate Mental Health Help-Seeking by Young Adults: Randomized Controlled Trial. J Med Internet Res.

[18] Andersen S, Harrison GW, Lau MI, Rutström EE (2018). Multiattribute utility theory, intertemporal utility, and correlation aversion. Int Econ Rev.

[19] Andersen S, Harrison GW, Lau MI, Rutström EE (2018). Multiattribute utility theory, intertemporal utility, and correlation aversion. Int Econ Rev.

[20] Zhang X, Guo X, Lai K, Guo F, Li C (2014). Understanding gender differences in m-health adoption: a modified theory of reasoned action model. Telemed J E Health.

[21] Reuter T, Ziegelmann JP, Wiedemann AU, Lippke S, Schüz B, Aiken LS (2010). Planning bridges the intention–behaviour gap: Age makes a difference and strategy use explains why. Psychol Health.

[22] Ng T, Feldman D (2009). How broadly does education contribute to job performance?. Pers Psychol.

[23] Verberne S, Batenburg A, Sanders R, van Eenbergen M, Das E, Lambooij MS (2019). Analyzing Empowerment Processes Among Cancer Patients in an Online Community: A Text Mining Approach. JMIR Cancer.

[24] Hargreaves S, Bath P, Duffin S (2018). Sharing and empathy in digital spaces: qualitative study of online health forums for breast cancer and motor neuron disease (amyotrophic lateral sclerosis). J Med Internet Res.

[25] Zhou J (2018). Factors Influencing People’s Personal Information Disclosure Behaviors in Online Health Communities: A Pilot Study. Asia Pac J Public Health.

[26] Zhang J (2015). Empirical research on information acquiring and information sharing intention and behavior in virtual communities. Inform Sci.

[27] Gui P, Hu X-F (2017). Study on factors influencing knowledge sharing willingness of health online community members based on internet word-of-mouth social exchange theory. J Modern Inf.

[28] Walker DM, Johnson T, Ford EW, Huerta TR (2017). Trust Me, I'm a Doctor: Examining Changes in How Privacy Concerns Affect Patient Withholding Behavior. J Med Internet Res.

[29] Deng Z, Hong Z, Ren C, Zhang Wei, Xiang Fei (2018). What Predicts Patients' Adoption Intention Toward mHealth Services in China: Empirical Study. JMIR Mhealth Uhealth.

[30] Abdelhamid M, Gaia J, Sanders GL (2017). Putting the Focus Back on the Patient: How Privacy Concerns Affect Personal Health Information Sharing Intentions. J Med Internet Res.

[31] Zhang X, Liu S, Chen X, Wang L, Gao B, Zhu Q (2018). Health information privacy concerns, antecedents, and information disclosure intention in online health communities. Inform Manag.

[32] Shen N, Sequeira L, Silver M, Carter-Langford A, Strauss J, Wiljer D (2019). Patient Privacy Perspectives on Health Information Exchange in a Mental Health Context: Qualitative Study. JMIR Ment Health.

[33] Holloway IW, Winder TJA, Lea ICH, Tan Diane, Boyd Donte, Novak David (2017). Technology Use and Preferences for Mobile Phone-Based HIV Prevention and Treatment Among Black Young Men Who Have Sex With Men: Exploratory Research. JMIR Mhealth Uhealth.

[34] Zhang X, Chen X, Hou D-L (2016). An analysis of online health information disclosure willingness influencing factors: An integrated model of TPB and privacy calculus. Information and Documentation Services. Inform Document Serv.

[35] Esmaeilzadeh P, Mirzaei T (2019). The Potential of Blockchain Technology for Health Information Exchange: Experimental Study From Patients' Perspectives. J Med Internet Res.

[36] Frost J, Vermeulen IE, Beekers N (2014). Anonymity versus privacy: selective information sharing in online cancer communities. J Med Internet Res.

[37] Kim D, Park K, Park Y, Ahn J-H (2019). Willingness to provide personal information: Perspective of privacy calculus in IoT services. Comput Hum Behav.

[38] Feri F, Giannetti C, Jentzsch N (2016). Disclosure of personal information under risk of privacy shocks. J Econ Behav Org.

[39] Househ M, Grainger R, Petersen C, Bamidis P, Merolli M (2018). Balancing Between Privacy and Patient Needs for Health Information in the Age of Participatory Health and Social Media: A Scoping Review. Yearb Med Inform.

[40] Dinev T, Hart P (2006). An Extended Privacy Calculus Model for E-Commerce Transactions. Inform Syst Res.

[41] Okazaki S, Li H, Hirose M (2009). Consumer Privacy Concerns and Preference for Degree of Regulatory Control. J Advert.

[42] Rahim FA, Ismail Z, Samy GN (2014). Information Privacy Concerns in the Use of Social Media Among Healthcare Practitioners: A Systematic Literature Review. Adv Sci Lett.

[43] Ozdemir ZD, Jeff Smith H, Benamati JH (2018). Antecedents and outcomes of information privacy concerns in a peer context: An exploratory study. Eur J Inform Syst.

[44] Bansal G, Zahedi F?, Gefen D (2010). The impact of personal dispositions on information sensitivity, privacy concern and trust in disclosing health information online. Decis Support Syst.

[45] Tisnado DM, Adams JL, Liu H, Damberg CL, Hu FA, Chen W, Carlisle DM, Mangione CM, Kahn KL (2006). Does the concordance between medical records and patient self-report vary with patient characteristics?. Health Serv Outcomes Res Method.

[46] Reisner SL, Randazzo RK, White Hughto JM, Peitzmeier S, DuBois LZ, Pardee DJ, Marrow E, McLean S, Potter J (2017). Sensitive Health Topics With Underserved Patient Populations: Methodological Considerations for Online Focus Group Discussions. Qual Health Res.

[47] Bansal G, Zahedi F, Gefen D (2017). The role of privacy assurance mechanisms in building trust and the moderating role of privacy concern. Eur J Inf Syst.

[48] Meng F, Guo X, Peng Z, Lai K, Zhao X (2019). Investigating the Adoption of Mobile Health Services by Elderly Users: Trust Transfer Model and Survey Study. JMIR Mhealth Uhealth.

[49] Bansal G, Zahedi F?, Gefen D (2010). The impact of personal dispositions on information sensitivity, privacy concern and trust in disclosing health information online. Decision Support Systems.

[50] Liang T, Ho Y, Li Y, Turban E (2014). What Drives Social Commerce: The Role of Social Support and Relationship Quality. Int J Electron Commer.

